# Facile Synthesis of SrCO_3_-Sr(OH)_2_/PPy Nanocomposite with Enhanced Photocatalytic Activity under Visible Light

**DOI:** 10.3390/ma9010030

**Published:** 2016-01-06

**Authors:** Alfredo Márquez-Herrera, Victor Manuel Ovando-Medina, Blanca Estela Castillo-Reyes, Martin Zapata-Torres, Miguel Meléndez-Lira, Jaquelina González-Castañeda

**Affiliations:** 1 Departamento de Ingeniería Agrícola, DICIVA, Campus Irapuato-Salamanca, Universidad de Guanajuato, Ex Hacienda el Copal, Carr. Irapuato-Silao km 9, Irapuato Gto 36500, Mexico; 2 Ingeniería Química, COARA, Universidad Autónoma de San Luis Potosí, Carr. a Cedral Km 5+600, San José de las Trojes, Matehuala, San Luis Potosí 78700, Mexico; victor.ovando@uaslp.mx (V.M.O.-M.); becr_iq@yahoo.com.mx (B.E.C.-R.); 3 Centro de Investigación en Ciencia Aplicada y Tecnología Avanzada, Unidad Legaría IPN, Calzada Legaría 694, Col. Irrigación, México D.F. 11500, Mexico; mzapatat@ipn.mx; 4 Departamento de Física, CINVESTAV-IPN, Apartado Postal 14-740, México D.F. 07000, Mexico; mlira@fis.cinvestav.mx; 5 Departamento de Ingeniería Ambiental, DICIVA, Campus Irapuato-Salamanca, Universidad de Guanajuato, Ex Hacienda el Copal, Carr. Irapuato-Silao km 9, Irapuato Gto 36500, Mexico; jaquegc1@hotmail.com

**Keywords:** composite materials, inorganic compounds, nanostructures, chemical synthesis, X-ray photo-emission spectroscopy (XPS)

## Abstract

Pyrrole monomer was chemically polymerized onto SrCO_3_-Sr(OH)_2_ powders to obtain SrCO_3_-Sr(OH)_2_/polypyrrole nanocomposite to be used as a candidate for photocatalytic degradation of methylene blue dye (MB). The material was characterized by Fourier transform infrared (FTIR) spectroscopy, UV/Vis spectroscopy, and X-ray diffraction (XRD). It was observed from transmission electronic microscopy (TEM) analysis that the reported synthesis route allows the production of SrCO_3_-Sr(OH)_2_ nanoparticles with particle size below 100 nm which were embedded within a semiconducting polypyrrole matrix (PPy). The SrCO_3_-Sr(OH)_2_ and SrCO_3_-Sr(OH)_2_/PPy nanocomposites were tested in the photodegradation of MB dye under visible light irradiation. Also, the effects of MB dye initial concentration and the catalyst load on photodegradation efficiency were studied and discussed. Under the same conditions, the efficiency of photodegradation of MB employing the SrCO_3_-Sr(OH)_2_/PPy nanocomposite increases as compared with that obtained employing the SrCO_3_-Sr(OH)_2_ nanocomposite.

## 1. Introduction

During the past decade, photocatalytic degradation has proven to be a promising technology for the removal of various organic pollutants in waste water because of its many attractive advantages, including its environmental friendly feature, relatively low cost, and low energy consumption [1,2,3,4,5,6,7,8,9,10]. Photocatalytic processes are methods that utilize the solar radiation energy to perform catalytic processes such as water splitting, waste mineralization, recovery of precious metals, *etc.* [11,12]. Many photocatalytic materials have wide bad-gap values and require ultraviolet light (UV) to be photoactive. However, the need of UV light for activating the photocatalyst greatly limits practical applications because of the low content of UV light in the solar spectrum (about 4%) [13]. Therefore to take complete advantage of the sunlight one needs to make a visible light activated photocatalyst or increase its efficiency in the UV light region. In order to narrow the band gap of these materials, several researchers have focused on modifications by doping with appropriate ions [6,14,15,16,17,18,19,20,21]. Also, it has been reported that by using composite films or powders consisting of two semiconducting photocatalysts the absorption edge is shifted to the visible light region, e.g., TiO_2_-SrTiO_3-δ_ [22], BiVO_4_-SrTiO_3_:Rh [23], Ag_3_PO_4_-Cr-SrTiO_3_ [24], Fe_2_O_3_-SrTiO_3_ [25], SrCO_3_-SrTiO_3_ [26], TiO_2_-SO_4_ [27], *g*-C_3_N_4_/Fe_3_O_4_/Ag_3_VO_4_ [28].

Conducting polymers (e.g., polyaniline, polypyrrole, and polythiophene) with delocalized conjugated structures have been widely studied due to their rapid photoinduced charge separation and relatively slow charge recombination [29,30]. In particular, polypyrrole (PPy) with extended *p*-conjugated electron systems has recently shown great promises to enhance photocatalytic activity owing to its unique electrical and optical properties, such as high absorption coefficients in the visible light, high mobility of charge carriers, and excellent stability [31,32]. Furthermore, PPy is also an efficient electron donor and good hole transporter upon visible light excitation. It was proposed that polypyrrole has the ability to channel the photoinduced holes from the surface of the semiconductor to the polymer/solution interface at a fast rate, which can then oxidize the pollutants [33,34]. The photocatalytic activity of semiconductors modified with PPy have shown that PPy can effectively enhance the photoactivity of TiO_2_ [35,36,37], Ag-TiO_2_ [38], Bi_2_WO_6_ [39], Fe_3_O_4_/ZnO [40], Bi_2_O_2_CO_3_ [41], *etc*.

Taking into account some reports about SrCO_3_-Sr(OH)_2_ composite as background [42,43,44,45,46,47], and due the SrCO_3_-Sr(OH)_2_/PPy nanocomposite has not been studied as a photocatalyst candidate, this manuscript describes a modified strategy for the preparation of SrCO_3_-Sr(OH)_2_ nanocomposite, followed by coating with the semiconducting polypyrrole (PPy) to increase its photoactivity in the visible light range. Both SrCO_3_-Sr(OH)_2_ and SrCO_3_-Sr(OH)_2_/PPy nanocomposites were tested for photodegradation of MB dye under visible light irradiation. The effects on MB dye initial concentration and the catalyst load on photodegradation efficiency were studied and discussed.

## 2. Results and Discussion

The process here described to obtain SrCO_3_-Sr(OH)_2_/PPy nanocomposite consists of two straightforward steps. The first step implies the production of Sr(OH)_2_ powders as a water insoluble white dust, which precipitates from the reaction medium according to the double-displacement chemical reaction in which the hydrated form of Sr(OH)_2_ can be formed.

In the search to find a cheap and straight route to obtain the SrCO_3_ phase, it was chose to dry Sr(OH)_2_ at ambient atmosphere to take advantage of the reaction with CO_2_ present in the air [42,43,44,45,46,47].

The second step implied the chemical polymerization of pyrrole monomer dispersing SrCO_3_-Sr(OH)_2_ nanocomposite using sodium dodecyl sulfate (SDS) surfactant. Since a practical point of view, the SrCO_3_-Sr(OH)_2_/PPy nanocomposite can be easily removed from MB aqueous solutions due to its water insolubility, facilitating its recovery. The most interesting characteristic of the SrCO_3_-Sr(OH)_2_/PPy composite is its high photoactivity under visible light as will be discussed later.

### 2.1. Characterization

#### 2.1.1. Chemical Composition

**Figure 1:**
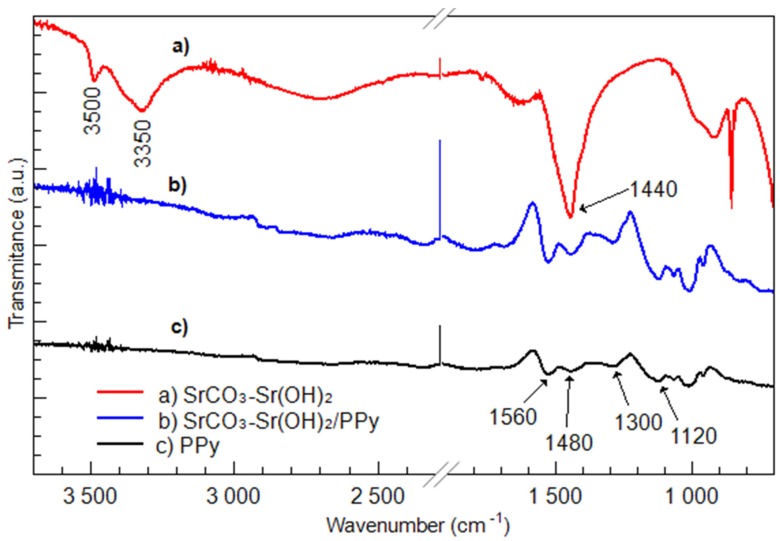
Fourier transform infrared (FTIR) spectra of (**a**) SrCO_3_-Sr(OH)_2_; (**b**) SrCO_3_-Sr(OH)_2_/PPy nanocomposites; and the characteristic signals of (**c**) polypyrrole matrix (PPy) chains.

Figure 1 shows the FTIR spectra of SrCO_3_-Sr(OH)_2_ and SrCO_3_-Sr(OH)_2_/PPy nanocomposites, also it can be observed the characteristic signals of PPy chains. The peak at 1480 cm^−1^ is ascribed to C–C ring stretching; the peak around 1560 cm^−1^ is due to C=C backbone stretching; and the peaks at 1300 and 1120 cm^−1^ are due to C–H in-plane and C–N stretching vibrations, respectively [48]. The peak located at 1560 cm^−1^ is considered as a reflection of the conducting polymer. Combined signals of SrCO_3_-Sr(OH)_2_/PPy were observed in Figure 1b indicating the interaction of SrCO_3_, Sr(OH)_2_ and PPy in the composite. The spectrum corresponding to SrCO_3_-Sr(OH)_2_ nanocomposite shows three main peaks, Figure 1a. The Peak at 3350 cm^−1^ is due to O–H physically adsorbed on the surface, the signal at 3500 cm^−1^ is ascribed to O–H bonds in Sr(OH)_2_ phase. The peak at 1440 cm^−1^ is usually observed when C=O bonds are present [46]; in our case this signal can be due to the presence of the SrCO_3_ phase. When the SrCO_3_-Sr(OH)_2_ nanocomposite is coated with PPy, the signals corresponding to SrCO_3_-Sr(OH)_2_ are masked by the semiconducting PPy, Figure 1b.

**Figure 2:**
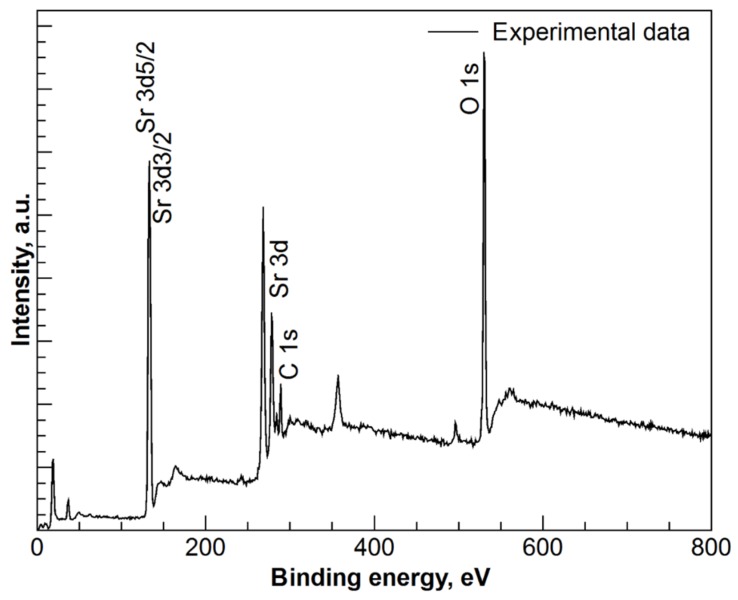
X-ray Photoelectron Spectroscopy (XPS) spectrum for SrCO_3_-Sr(HO)_2_ nanocomposite.

In order to obtain insights into the chemical environment of the elements of the SrCO_3_-Sr(OH)_2_ powders a study using X-ray photoelectron spectroscopy (XPS) was performed. The general survey XPS spectrum, Figure 2, shown peaks related with Sr, O and C.

**Figure 3:**
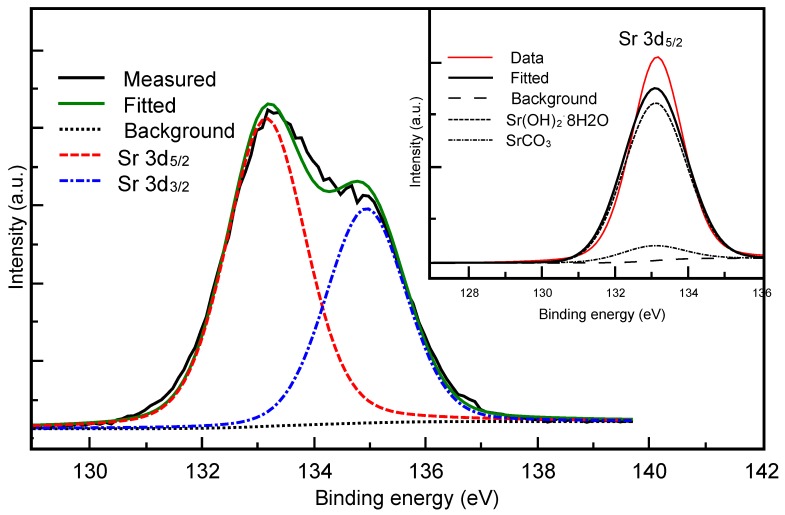
XPS spectrum of Sr 3d_5/2_ and Sr 3d_3/2_ for the SrCO_3_-Sr(HO)_2_ sample. The inset shows the deconvolution of the Sr 3d_5/2_ signal.

Figure 3 shows the high resolution XPS spectrum associated to the Sr binding energies for the SrCO_3_-Sr(OH)_2_ sample. It shows a peak fit analysis of the Sr 3d_5/2_ and Sr 3d_3/2_ signals with mixed Gaussian-Lorentzian profiles that reveals two underlying components of the binding energies at 133.2 eV and 134.8 eV. The inset in Figure 3 shows only the deconvolution of the Sr 3d_5/2_ signal that is attributed to Sr bonded to Sr(HO)_2_·8H_2_O and SrCO_3_. The deconvolution for the Sr 3d_3/2_ signal was not carried out because there is no reported information about its strength in the compound Sr(OH)_2_. The positions of the peaks were obtained from the X-ray Photoelectron Spectroscopy Database of NIST [49]. This result confirms that Sr(HO)_2_·8H_2_O and SrCO_3_ phases are present in the composite.

#### 2.1.2. Crystallinity and Morphology

**Figure 4:**
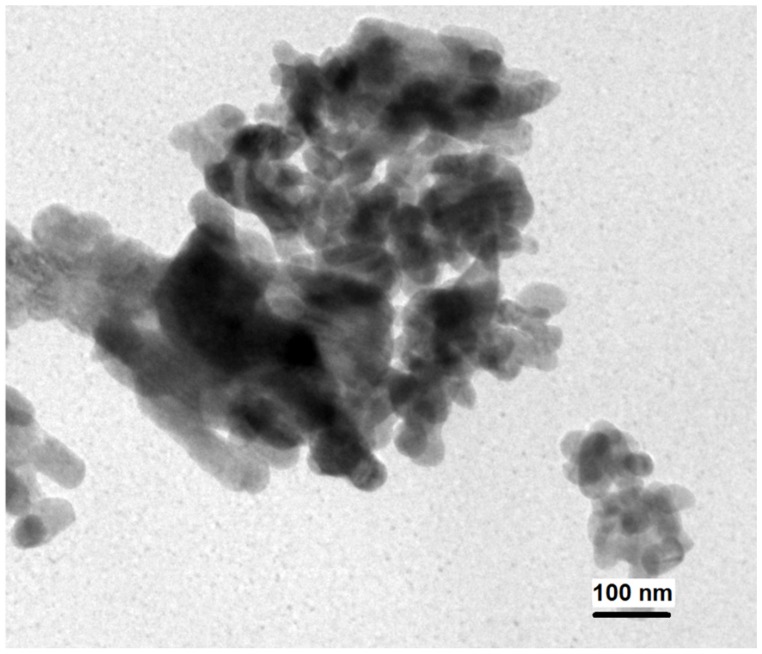
Transmission electron microscopy (TEM) image of the as-prepared SrCO_3_-Sr(OH)_2_ nanoparticles without polypyrrole.

Figure 4 shows the transmission electron micrograph corresponding to SrCO_3_-Sr(OH)_2_ sample without polypyrrole. As can be seen, the powders consisted of clusters of SrCO_3_-Sr(OH)_2_ nanoparticles. Although the morphology of nanoparticles is not clearly defined, it looks like circular shapes. It can be observed that the particle size is below 100 nm. Due TEM technique burns the polypyrrole, it is not appropriate to verify the existence of PPy on the surface of SrCO_3_-Sr(OH)_2_ sample with TEM images. However it is worth to mentioning that the composite SrCO_3_-Sr(OH)_2_/PPy has a core-shell structure [50].

BET area for SrCO_3_-Sr(OH)_2_ particles was found to be 8.5 m^2^/g, this high surface area is already evident from TEM image. This value is similar to the reported by Viriya-Empikul, *et al.* [51] for SrCO_3_-Sr(OH)_2_·H_2_O composite (5.2 m^2^/g). This high surface area has a relevance because the surface of the photocatalytic material in contact with the contaminant plays an important role in determining the photocatalytic activity of the composite powders [52].

**Figure 5:**
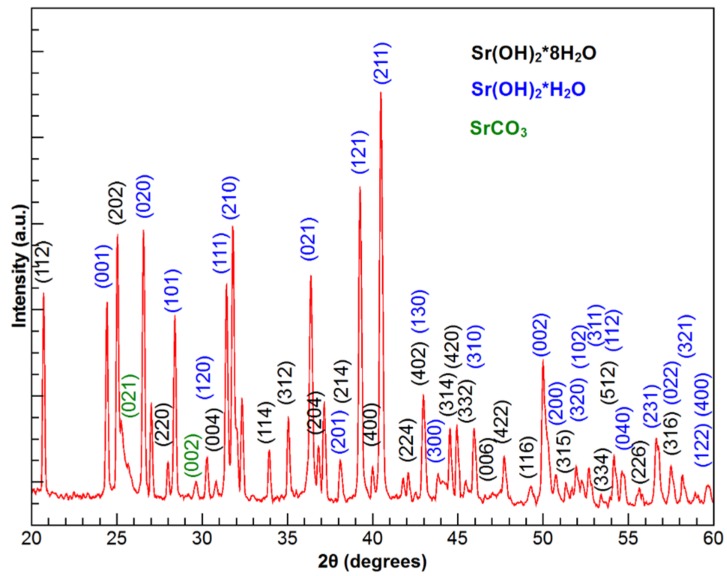
The X-ray diffraction (XRD) pattern obtained for the SrCO_3_-Sr(OH)_2_ nanocomposite.

Figure 5 shows X-ray diffractogram of the SrCO_3_-Sr(OH)_2_ nanocomposite; the corresponding to the SrCO_3_-Sr(OH)_2_/PPy composite just incorporated a broad signal characteristic of amorphous polypyrrole. The positions of the diffraction peaks associated to the orthorhombic Sr(OH)_2_∙8H_2_O, Sr(OH)_2_∙H_2_O and SrCO_3_ from the 271438, 281222 and 050418 cards of the Powder Diffraction File database (PDF card) are also shown. The close coincidence with the reported positions allows to establish that the peaks presented in the experimental diffractogram are due to the diffraction from the planes of the Sr(OH)_2_∙H_2_O and Sr(OH)_2_∙8H_2_O. A small signal from the planes of the SrCO_3_ phase was found. The X-ray diffraction analysis corroborates that the powders are composed by Sr(OH)_2_∙H_2_O, Sr(OH)_2_∙8H_2_O and SrCO_3_ (called SrCO_3_-Sr(OH)_2_) highly ordered crystals because there is a complete correspondence between the experimental diffraction peaks and the data base positions (for annealed sample see Figure S1).

From Rietveld refinement with an adjust factor *R*_WP_ better than 10%, the percentage of phases found in the composite were 59.3% ± 1.2%, 32.3% ± 0.7% and 8.4% ± 0.3% for Sr(OH)_2_∙8H_2_O, Sr(OH)_2_∙H_2_O and SrCO_3_, respectively.

#### 2.1.3. Photocatalytic Activity

**Figure 6:**
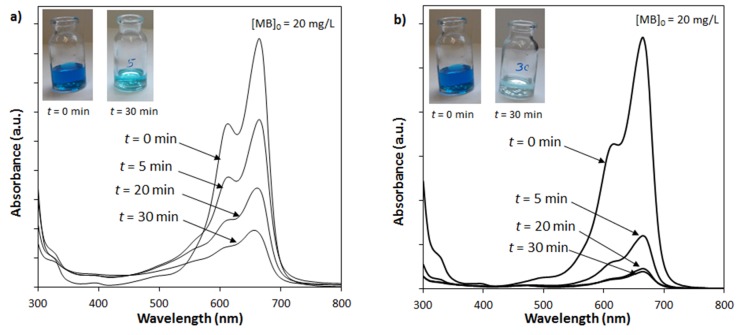
Ultraviolet-visible (UV/Vis) spectra of methylene blue dye (MB) aqueous solutions at different times for a 0.2 g of (**a**) SrCO_3_-Sr(OH)_2_; and (**b**) SrCO_3_-Sr(OH)_2_/PPy nanocomposites.

The photocatalytic performances of the SrCO_3_-Sr(OH)_2_ and Sr(OH)_2_/PPy nanocomposites were studied following the degradation process of aqueous solutions of MB dye under visible light irradiation. Figure 6 shows the UV/Vis spectra of MB aqueous solutions at different times. Solutions were prepared employing an initial MB concentration of 20 mg/L and 0.2 g of both (a) SrCO_3_-Sr(OH)_2_ and (b) SrCO_3_-Sr(OH)_2_/PPy nanocomposite load. It should be noted that there is a small difference in the SrCO_3_-Sr(OH)_2_ weight of the catalyst employed because the PPy. However, it highlights the positive effect of PPy on the photocatalytic activity of SrCO_3_-Sr(OH)_2_. It can be seen that the peak at λ = 665 nm decreases with the visible light irradiation time, reaching a minimum after 30 min for the SrCO_3_-Sr(OH)_2_/PPy nanocomposite. Insets in Figure 6 clearly shown a more discolored solution for the catalyst containing PPy. Based in the above results, photodegrading kinetics studies were made considering 30 min of reaction.

**Figure 7:**
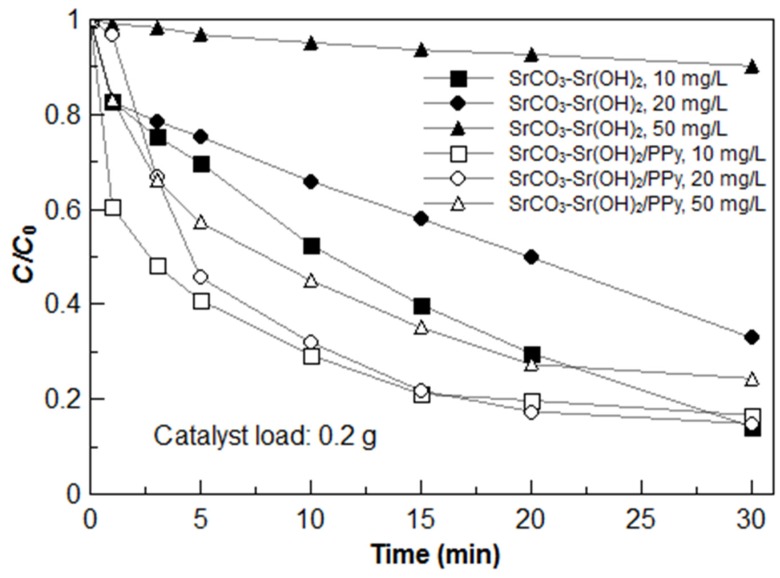
Kinetics of MB dye photodegradation under visible light irradiation using SrCO_3_-Sr(OH)_2_ and SrCO_3_-Sr(OH)_2_/PPy nanocomposites for the MB initial concentrations indicated in the label. The catalyst load was 0.2 g for each case.

Figure 7 shows the ratio of residual to initial MB concentration (*C*/*C_0_*) as a function of time using the SrCO_3_-Sr(OH)_2_ and the SrCO_3_-Sr(OH)_2_/PPy nanocomposites (0.2 g for both cases) at different MB initial concentrations. It can be observed that for the lower value of MB initial concentration, similar degradation efficiencies can be achieved after 30 min of visible light exposition for both materials (efficiency around 85%). However when the MB initial concentration was increased up to 50 mg/L, maintaining constant the catalyst load to 0.2 g, lower degradation efficiencies were obtained; 9.8% for bare SrCO_3_-Sr(OH)_2_ nanocomposite compared to 75.6% for SrCO_3_-Sr(OH)_2_/PPy nanocomposite. Furthermore, only 10 min were needed to achieve 71% of degradation for 10 mg/L of initial MB concentration using the SrCO_3_-Sr(OH)_2_/PPy nanocomposite.

**Figure 8:**
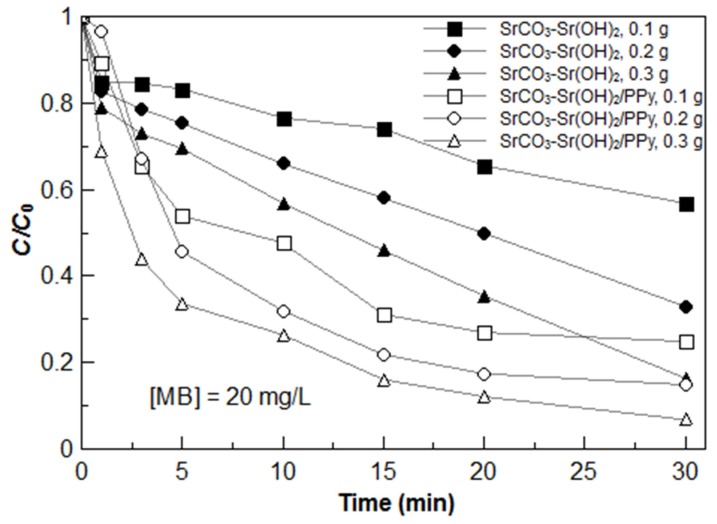
MB dye photodegradation kinetics under visible light irradiation using 20 mg/L of MB initial concentration and different SrCO_3_-Sr(OH)_2_ and SrCO_3_-Sr(OH)_2_/PPy nanocomposites loading.

By other hand, Figure 8 shows the effect of SrCO_3_-Sr(OH)_2_ and SrCO_3_-Sr(OH)_2_/PPy nanocomposite photocatalyst load on the photodegradation kinetics with a fixed initial MB concentration (20 mg/L). Employing 0.3 g of catalyst load, it can be observed for the SrCO_3_-Sr(OH)_2_ nanocomposite that 43.1% of degradation efficiency was achieved after 10 min of visible light irradiation and 83.6% after 30 min, whereas for the SrCO_3_-Sr(OH)_2_/PPy nanocomposite the corresponding efficiencies were 73.6% and 93.2%, respectively. Decreasing the amount of catalyst to 0.1 g and after 30 min of degradation, it resulted in a decrease in degradation efficiency from 93.2% to 75.1% for the SrCO_3_-Sr(OH)_2_/PPy nanocomposite; while for SrCO_3_-Sr(OH)_2_ nanocomposite dropped the degradation efficiency to 43.1%, thus, the photocatalyst amount strongly affects the efficiency of MB degradation, and show the enhanced performance of the SrCO_3_-Sr(OH)_2_/PPy nanocomposite under the studied conditions. These results show that SrCO_3_-Sr(OH)_2_ based nanocomposites are promising materials with excellent performance in photocatalytic applications, and the incorporation of PPy enhances noticeably their performance.

Blank measurements were carried out employing both catalysts and MB solutions at low concentrations without observe any degradation at all confirming that these catalysts are activated by visible light (Figures S2–S4).

Because Sr(OH)_2_ is an ionic compound, not a semiconductor, the photocatalytic activity of the composite containing Sr(OH)_2_ cannot explained by changes in the band structure of it [52]. It is possible that the presence of the SrCO_3_ phase is the responsible of the improvement in the photocatalytic activity of the composite [26]. The increase in carriers due to the absorption process in the semiconductor PPy coating injects more electrons to the SrCO_3_ compound [48,53,54] increasing its photodegradation activity efficiency. However, the principle of the photocatalytic oxidation due to the Sr(OH)_2_ phase still needs to be clarified.

**Figure 9:**
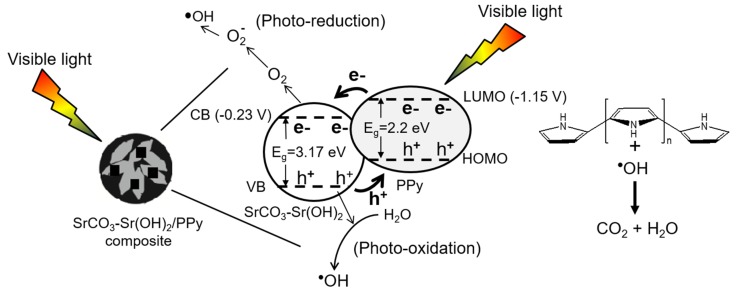
Possible MB dye photodegradation process.

In our particular case, the formation of semiconducting SrCO_3_ (which has a reported band gap energy of 3.17 eV) [54,55] during both, drying of pure Sr(OH)_2_ and dye photodegradation due to the CO_2_ adsorption from air and water, respectively, permits the explanation of dye photodegradation mechanism as shown in the Figure 9. The performance of this composite is determined by the relative positions of the bands of nanoparticles and PPy. The values of each bandgap are reported in Figure 9, however, the exact determination of their position is beyond the scope of this work.

When visible light impinges on the composite surface, electrons are promoted from HOMO to LUMO of PPy (which has a reported band gap energy of 2.2 eV) [56], generating holes in the PPy chain (h+), electrons in the LUMO (−1.15 V *versus* NHE) [56], travel through PPy chains to conduction band (CB), (−0.23 V *versus* NHE) [57], of inorganic material, which can react with oxygen solved in the aqueous phase initiating photo-reduction. On the other hand, electrons in the valence band (VB) of inorganic material travels to h+ in the HOMO of PPy, generating a hole in the VB in the inorganic material. These holes can react with water generating ·OH radicals, which attack organic molecules (photo-oxidation) until mineralization is done.

In the photocatalytic degradation of methylene blue, not only do $O_{2}^{-}$ and ·OH play important roles, but the holes generated in the HOMO band of PPy also play a role, however they have a lower oxidative capability than those in the valence band of SrCO_3_, as shown in Figure 9. It is energetically unfavorable to use pure PPy to oxidize methylene blue molecules to form ·OH radicals, because the methylene blue molecules need to be attacked by hydroxyl radicals to generate organic radicals or other intermediates.

## 3. Materials and Methods

### 3.1. Materials

In the present study, all chemicals used were analytical reagent grade. Strontium nitrate hexahydrate (Sr(NO_3_)_2_∙6H_2_O) and sodium hydroxide (NaOH) were purchased from Onyx-Met, Inc. (Olsztyn, Poland). Methylene blue dye was purchased from Fluka (Toluca, Mexico). Pyrrole monomer and ammonium persulfate (APS) were purchased from Sigma-Aldrich (Toluca, Mexico). Sodium dodecyl sulfate (SDS) was acquired from Hycel (Guadalajara, Mexico). Deionized water was used in all the experiments.

### 3.2. Methods

#### 3.2.1. Synthesis of SrCO_3_-Sr(OH)_2_/PPy Nanocomposite

**Figure 10:**
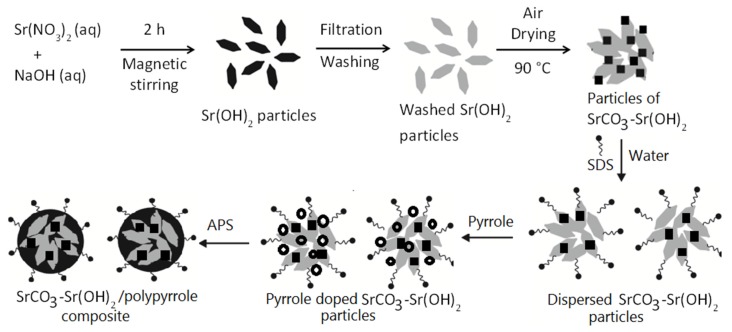
Experimental process to obtain SrCO3-Sr(OH)_2_/PPy nanocomposite.

The SrCO_3_-Sr(OH)_2_/PPy nanocomposite was prepared as described in Figure 10: first, NaOH (2.0000 g) and Sr(NO_3_)_2_∙6H_2_O (10.5814 g) were mixed together in distilled water (30 mL) (molar ratio of NaOH/Sr(NO_3_)_2_ of 2:1) under 450 rpm magnetic stirring by 2 h, resulting in a precipitate as a fine white powder of Sr(OH)_2_ which was water insoluble. Afterward, the precipitates were filtered using Whatman 42 filter paper, washed several times with de-ionized water. Then, sample was dried at 90 °C in air for 2 h without annealing (Figures S5 and S6). Afterwards, dried sample of SrCO_3_-Sr(OH)_2_ (0.2500 g) was well dispersed in an aqueous solution of SDS (consisting in 30 mL of water and 0.8 g of SDS). This mixture was ultrasonicated (Cole-Parmer Instruments, CPX 130, Vermon Hill, IL, USA) by 10 min for homogenization; 0.4 g of pyrrole monomer was added and homogenized under magnetic stirring through 2 h. Then, APS was dissolved in 10 mL of water (0.6 M) and added to the reaction mixture to start pyrrole polymerization. The reaction proceeded under magnetic stirring for 1 h. The reaction mixture was poured into an excess of methanol to precipitate the SrCO_3_-Sr(OH)_2_/PPy composite (black dust). The sample was decanted and dried at 60 °C in an oven for 24 h.

#### 3.2.2. Characterization

The chemical environment structures of strontium, carbon and oxygen were analyzed by X-ray Photoelectron Spectroscopy (XPS) (model K alpha, Thermo Scientific, Waltham, CT, USA). The general survey, as well as the high resolution spectra in the regions of the C 1s, O 1s and Sr 3d were obtained. The binding energy of the C 1s line at 284.5 eV was taken as the reference peak to calibrate the obtained spectra. The background subtraction was performed using the mathematical model derived by Shirley [58]. The Sr signal curve was fitted with an asymmetric Gaussian-Lorentzian function. The X-ray diffraction (XRD) measurement was performed with a Rigaku X’pert diffractometer (Rigaku, Tokio, Japan) using the Cu_Kα_ line (λ_kα1_ = 1.54056 Å and λ_kα2_ = 1.54439 Å) and the correspondence between the experimental diffraction peaks and database position was made using the Match! 3 phase identification from powder diffraction software (Crystal Impact, Bonn, Germany). In order to determinate the percentage of each phase in the composite, the quantitative phase composition was analyzed according to the Rietveld refinement method [59] using the software Maud (University of Trento, Trento, Italy) [60]. The crystal data for each phase used in the quantitative phase analysis were obtained from Inorganic Crystal Structure Database (ICSD). By other hand, the particle size of representative SrCO_3_-Sr(OH)_2_ powders were observed via transmission electron microscopy (TEM) using a JEOL-2010 system (Jeol, Pleasanton, CA, USA) operated at 200 kV where the powders were dispersed in distilled water and deposited on carbon foil on copper grids. The particle size was calculated using the ImageJ 1.46c software (National Institute of Mental Health, Rockville, MD, USA) in TEM images. The average surface area (*S*_BET_) of the SrCO_3_-Sr(OH)_2_ particles was obtained using a Brunauer-Emmett-Teller (BET) method [61]. For measuring nitrogen adsorption, 68.7 mg of sample, was used. It was dehydrated for four hours at 200 °C, then the adsorption of nitrogen was measured at liquid nitrogen temperature (−197.392 °C).

#### 3.2.3. Photoactivity in the Visible Light of Synthesized Materials

The synthesized SrCO_3_-Sr(OH)_2_ and the SrCO_3_-Sr(OH)_2_/PPy nanocomposites were tested by photodegradation of aqueous solutions of MB dye under visible light irradiation. The reactor consisted of a glass vessel with two quartz bulbs, the first for water recirculation at constant temperature (20 °C) and the second to insert the visible light source. The effect of the catalyst load on MB degradation was studied using 0.1 g, 0.2 g and 0.3 g of SrCO_3_-Sr(OH)_2_ and SrCO_3_-Sr(OH)_2_/PPy nanocomposites; catalyst were mixed with 150 mL of aqueous solutions of MB at 20 mg/L of initial concentration (*C*_0_). Afterwards, the mixture was charged to the reactor. In each case, the tested solutions were exposed to a visible light source from a halogen lamp with tungsten filament (Philips LongLife EcoVision H7, 12 V, and 55 W) and a cutoff filter (λ > 400 nm). Aliquots of 1.5 mL were obtained at different times, centrifuged and poured into a quartz cuvette to determine UV/Vis spectra (250 nm to 800 nm of wavelength) and absorbance (Genesys 10, Thermo-Spectronic) at a wavelength of 664 nm to calculate residual MB concentrations (*C*) from a calibration curve. In addition, initial MB concentrations were varied from 10 mg/L to 50 mg/L when working with SrCO_3_-Sr(OH)_2_ and SrCO_3_-Sr(OH)_2_/PPy composites at a fixed load of 2.0 g/L.

## 4. Conclusions

On the basis of FTIR spectroscopy, XPS, XRD and TEM results, the successful synthesis of SrCO_3_-Sr(OH)_2_/PPy nanocomposite was obtained using Sr(NO_3_)_2_∙6H_2_O, NaOH, SDS and pyrrole monomer as precursors. The measurements indicate that the obtained material corresponds to SrCO_3_-Sr(OH)_2_ nanocomposite with particle size below 100 nm, which were immersed into a semiconducting polypyrrole matrix. The SrCO_3_-Sr(OH)_2_ particles showed only 9.7% of MB dye photodegradation after 30 min of visible light irradiation using a MB initial concentration of 50 mg/L and a catalyst load of 1.3 g/L of solution; and for the same conditions but with 20 mg/L of MB dye initial concentration, the efficiency was 67.0%. The corresponding efficiencies using the SrCO_3_-Sr(OH)_2_/PPy composite were 75.6% and 85.2%, respectively. It was also observed that using a catalyst load of 2.0 g/L of solution with 20 mg/L of MB dye initial concentration and after 30 min of photodegradation, 83.6% and 93.2% of efficiency were obtained for SrCO_3_-Sr(OH)_2_and SrCO_3_-Sr(OH)_2_/PPy nanocomposites, respectively. In summary, the results obtained in the present study indicate that SrCO_3_-Sr(OH)_2_/PPy nanocomposite increased the catalytic efficiency of SrCO_3_-Sr(OH)_2_ nanocomposite and it may serve as a promising efficient photocatalyst for the degradation of organic contaminants as the methylene blue. Also, it is important to note that this nanocomposite meets at least four requirements: easy preparation/synthesis with availability of the raw materials, low cost, and highly effective.

## Supplementary Materials

The following are available online at www.mdpi.com/1996-1944/9/1/30/s1.
