# Supplementary material for: Facile Synthesis of SrCO3-Sr(OH)2/PPy Nanocomposite with Enhanced Photocatalytic Activity under Visible Light
Source: Materials (Basel). 2016 Jan 6;9(1):30. doi: 10.3390/ma9010030 (PMC5456521; doi:10.3390/ma9010030)
Supplement: Supplementary file 1 [file materials-09-00030-s001.pdf]

Supplementary Materials: Facile Synthesis of $SrCO_3$-$Sr(OH)_2$/PPy Nanocomposite with Enhanced Photocatalytic Activity under Visible Light

Alfredo Márquez-Herrera, Victor Manuel Ovando-Medina, Blanca Estela Castillo-Reyes, Martin Zapata-Torres, Miguel Meléndez-Lira and Jaquelina González-Castañeda

Figure S1 shows X-ray diffractogram of the $SrCO_3$-$Sr(OH)_2$ nanocomposite without polypyrrole annealed at 700 °C during 4 h. The positions of the diffraction peaks associated to the orthorhombic $Sr(OH)_2{\cdot}H_2O$, $Sr(OH)_2$ and $SrCO_3$ from the 281222, 028848 and 050418 cards of the Powder Diffraction File database (PDF card) are also shown. The peaks presented are due to the diffraction from the $Sr(OH)_2{\cdot}H_2O$ and $Sr(OH)_2$ planes. A small signal from the $SrCO_3$ phase is observed. The X-ray diffraction analysis corroborates that the powders are composed by $Sr(OH)_2$, $Sr(OH)_2{\cdot}H_2O$ and $SrCO_3$ without $Sr(OH)_2{\cdot}8H_2O$ because there is a complete correspondence between the experimental diffraction peaks and the data base positions.

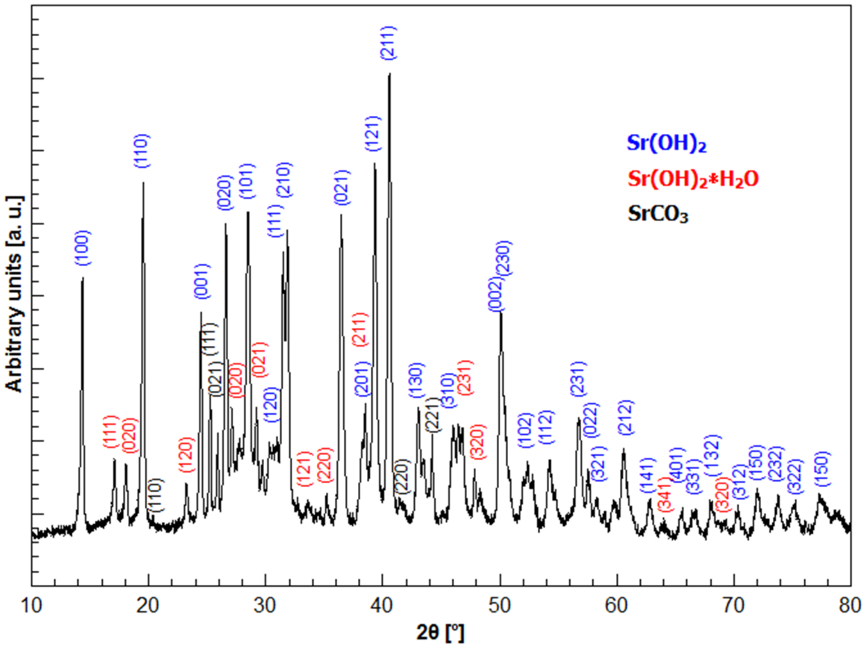

Figure S1. The XRD pattern of the $SrCO_3$-$Sr(OH)_2$ nanocomposite which was annealing at 700 °C during 4 h.

From Rietveld refinement with an adjust factor R_{WP} better than 7%, the percentage of phases found in composite were 54.7% ± 0.7%, 37.0% ± 0.1% and 8.3% ± 0.2% for $Sr(OH)_2{\cdot}H_2O$, $Sr(OH)_2$ and $SrCO_3$. This shows that the material is dehydrated (without $Sr(OH)_2{\cdot}8H_2O$) after the heat treatment.

Figure S2 shows the ratio of residual to initial MB concentration (C/C_0) as function of time using pure PPy (0.2 g) with 50 mg/L MB initial concentration in 150 mL MB aqueous solutions. It can be observed that a similar absorption was observed for the sample during 30 min of visible light exposition. The picture of the solutions at 0 min and 30 min of photodegradation time are shown in the inset in Figure S3, in which can be observed a similar coloration in samples.

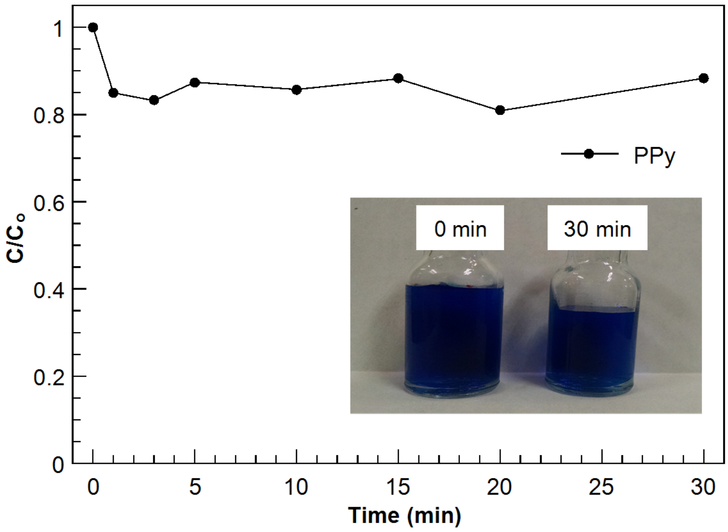

Figure S2. Kinetics of MB dye photodegradation under visible light irradiation using pure PPy (0.2 g). The inset shows the picture of the solutions at 0 min and 30 min of photodegradation.

Figure S3 shows the results of blank measurements for 50 mL solution (10 mg/L of initial MB concentration) using 0.2 g of $SrCO_3$-$Sr(OH)_2$ nanocomposite without PPy during 30 min.

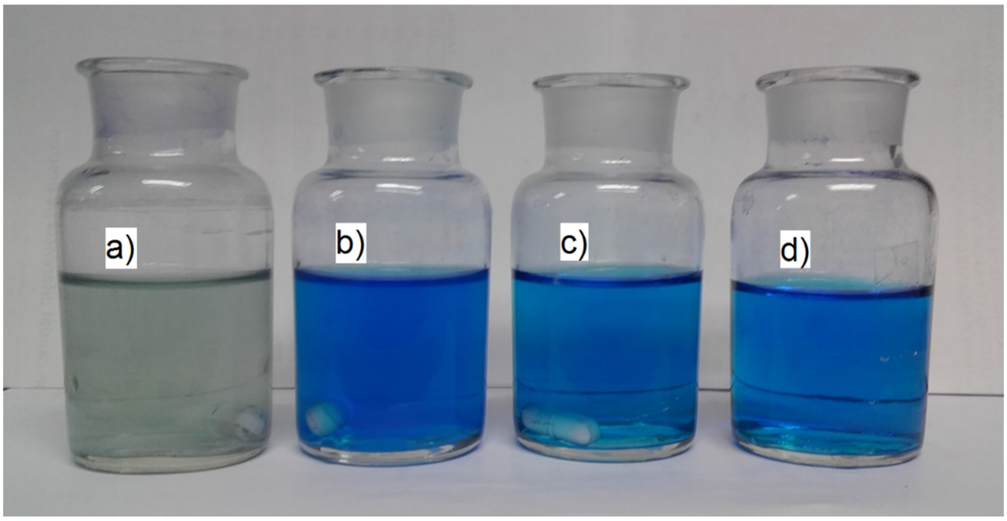

Figure S3. Picture of the MB solutions at 30 min of photodegradation time (**a**) with catalyst; and (**b**) without catalyst. Additionally, the MB solutions was place in the dark during 30 min (**c**) with catalyst; and (**d**) without catalyst.

Figure S4 shows the ratio of residual to initial MB concentration (C/C_0) as function of time using $SrCO_3$-$Sr(OH)_2$ and $SrCO_3$-$Sr(OH)_2$/PPy nanocomposite (0.2 g) at 50 mg/L MB initial concentration in 150 mL MB aqueous solution in the dark. It can be observed that a similar absorptions were observed for the samples during 30 min in the dark.

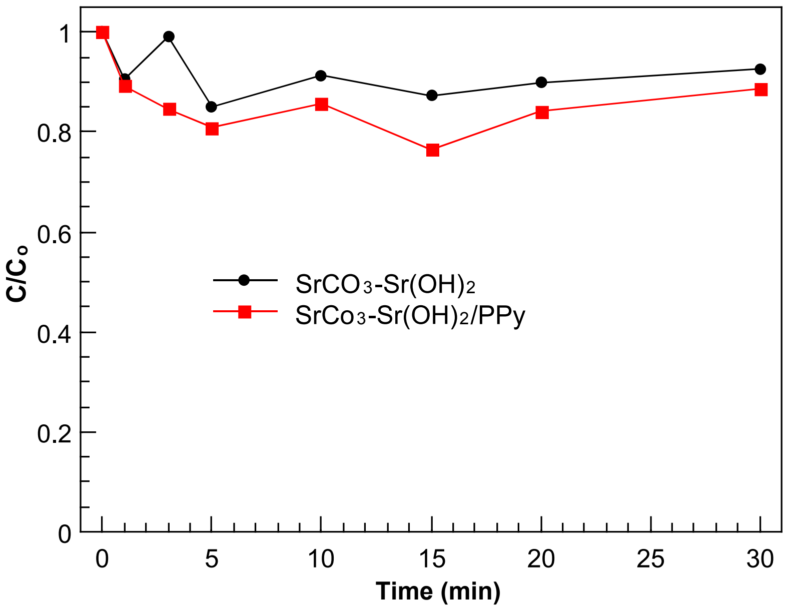

Figure S4. Kinetics of MB dye degradation under dark conditions using $SrCO_3$-$Sr(OH)_2$ and $SrCO_3$-$Sr(OH)_2$/PPy nanocomposite. The catalyst load was 0.2 g at 50 mg/L MB initial concentration in 150 mL MB aqueous solution.

Figure S5 shows the UV/Vis spectra of MB aqueous solutions at 30 min for a MB initial concentration of 10 mg/L and 0.1 g of $SrCO_3$-$Sr(OH)_2$ nanocomposite load (in 150 mL). $SrCO_3$-$Sr(OH)_2$ nanocomposites were annealed at 700 °C during 0 h, 2 h, 4 h, 6 h and 8 h. It can be seen that absorption peak at λ = 665 nm decreases for all samples. The composite without annealing presents the major photodegradation as reflected in the smaller intensity of the peak. The picture of the solutions at 30 min of photodegradation time are shown in the inset in Figure S1, in which can be observed that the best discoloration after 30 min corresponds to the sample without annealing (0 h). In the same way, kinetics studies of photodegrading were made with 30 min of reaction.

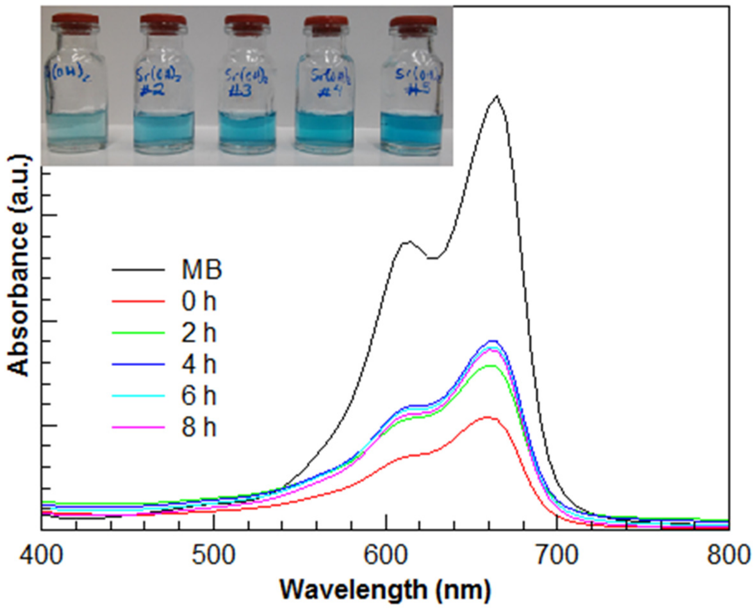

Figure S5. UV/Vis spectra of MB aqueous solutions with initial concentration of 10 mg/L at 30 min for 0.1 g of $SrCO_3$-$Sr(OH)_2$ nanocomposite annealed at different temperatures. The inset shows the picture of the solutions at 30 min of photodegradation. Containers are ordered according to the annealing time of the catalysts.

Figure S6 shows the ratio of residual to initial MB concentration (C/C_0) as function of time using the annealed $SrCO_3$-$Sr(OH)_2$ nanocomposite (0.1 g) with 10 mg/L MB initial concentration in 150 mL MB aqueous solutions. It can be observed that for the annealed samples a similar degradation efficiencies can be achieved after 30 min of visible light exposition. The higher degradation efficiency was observed employing the catalyst without annealing.

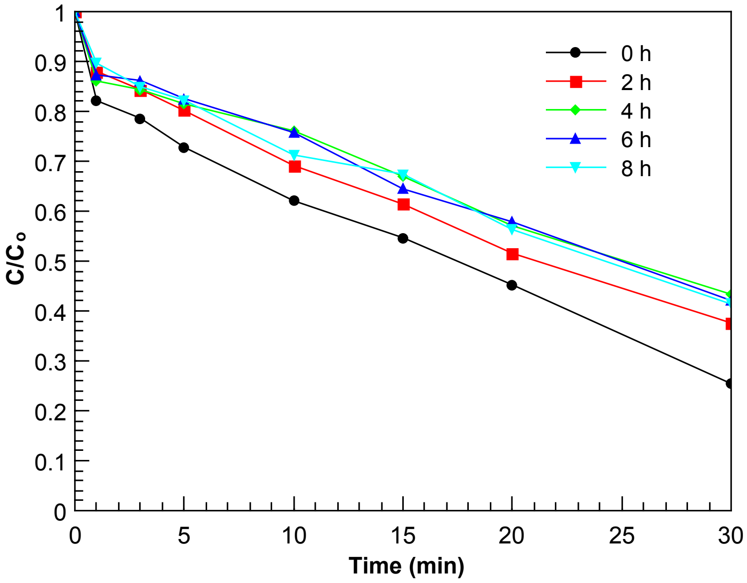

Figure S6. Kinetics of MB dye photodegradation under visible light irradiation using $SrCO_3$-$Sr(OH)_2$ nanocomposite annealed at 700 °C during different times. The catalyst load was 0.1 g for each case.
